# Epidermal Growth Factor Receptor Tyrosine Kinase Inhibitors for Elderly Patients with Advanced Non-Small Cell Lung Cancer

**DOI:** 10.1155/2010/348174

**Published:** 2010-06-20

**Authors:** F. Meriggi, A. Zaniboni

**Affiliations:** Oncology Department, Fondazione Poliambulanza, 25124 Brescia, Italy

## Abstract

Lung cancer is the leading cause of cancer-related mortality in both men and women and approximately 219,440 new cases of nonsmall cell lung cancer (NSCLC) were estimated to occur in the USA in 2009, which caused 159,390 NSCLC-related deaths. More than 50% of cases of advanced NSCLC are diagnosed in patients older than age 65, and recent Surveillance Epidemiology and End Results (SEERs) data suggest that the median age at diagnosis is 70 years. Until recently, the disease has been undertreated in this patient population, with a perception among many clinicians that elderly patients do not tolerate chemotherapy or radiotherapy. So, single agent chemotherapy is the recommended approach by the ASCO and International Expert Panels in unselected patients. The introduction of novel targeted therapies, such as Epidermal Growth Factor Receptor (EGFR) Tyrosine Kinase Inhibitors (TKIs) which improved survival versus placebo in patients who had previously failed on chemotherapy, gives clinicians new, effective, and better tolerated options to consider when treating NSCLC in elderly patients. This paper describes the advances of EGFR TKIs for elderly patients with advanced NSCLC.

## 1. Introduction

Lung Cancer is the most common cancer in the world and the leading cause of cancer-related deaths in Western countries [1]. NSCLC constitutes between 80% and 85% of all lung cancers. The median age at diagnosis is now 70 years [2] and most patients with NSCLC have incurable disease at diagnosis, with only approximately 15% presenting with localized disease [3]. Treatment for advanced disease is palliative in nature. In patients with a good performance status (PS), first-line treatment with platinum-based combination chemotherapy leads to improved overall survival (OS) and improvement in symptoms [4–6]. However, in elderly patients, single-agent chemotherapy with a third-generation agent (vinorelbine, gemcitabine, or taxanes) is the recommended approach by the American Society of Clinical Oncology guidelines and international expert panels in unselected patients [7, 8]. Moreover, in current practice, the elderly are often excluded from participation in clinical trials and receive empirical or inadequate treatment [9]. Retrospective analyses of trials not restricted to elderly patients have generally demonstrated that the elderly have similar response rates (RRs) to chemotherapy as younger patients and also similar survival benefits. Most studies also have shown that older patients are more likely to stop treatment as a result of toxicity, although, objectively, these studies have reported either little or no increase in toxicity in elderly subgroup [8, 10–16]. Availability of an effective, less toxic therapy might help extend potentially beneficial treatment to a greater proportion of older patients with advanced NSCLC and TKIs represent just these kinds of drugs. The EGFR family is part of a complex signal-transduction network that is central to several critical cellular processes. The EGFR (also known as ErbB-1/HER1) is a 170-kDa transmembrane glycoprotein that consists of an extracellular domain that recognizes and binds to specific ligands, a hydrophobic transmembrane domain, which is involved in interactions between two receptors within the cell membrane, and an intracellular domain that contains the tyrosine kinase enzymatic activity. Since EGFR expression is often found in NSCLC cells [17, 18], it has been the focus of efforts to develop new agents that target the EGFR pathway. Erlotinib and gefitinib inhibit the tyrosine kinase activity of EGFR and have been studied extensively [19–22]. Besides the two rather selective TKIs of EGFR, other TKIs with a broader spectrum of activity and other Monoclonal Antibodies (MoAb) to extracellular domain of the EGFR are also being tested in advanced NSCLC. Among broader spectrum EGFR TKIs are lapatinib, which are also active against ErbB2/neu, another member of the EGFR family of receptors, and vandetanib, which inhibits the Vascular Endotelial Growth Factor (VEGF) receptor [23]. However, lapatinib is approved for the treatment of advanced breast cancer and the development of vandetanib has been discontinued by AstraZeneca in 2010.

## 2. Gefitinib

Gefitinib (ZD1839) is an orally available EGFR small-molecule TKI. In two large phase II trials, IDEAL 1 and 2, gefitinib monotherapy was demonstrated to be active and well tolerated in advanced NSCLC patients which progressed after one or more chemotherapy regimens [20, 21]. These trials led to US FDA approval of gefitinib as salvage third-line therapy for NSCLC in May 2003, as a single agent after failure of both platinum-based and docetaxel chemotherapies. Gefitinib activity as a single agent at a dose of 250 mg was not confirmed in a placebo controlled randomized phase III Iressa Survival Evaluation in Lung Cancer (ISEL) trial in advanced NSCLC with heavily pretreated patients [24]. However, preplanned subgroup analyses indicated statistically different survival benefit in never smokers and in patients of Eastern Asian origin. In June 2005, on the basis of the lack of survival benefit in the ISEL study, the FDA restricted the use of gefitinib to patients participating in a clinical trial or who were continuing to benefit from treatment already initiated [25]. Currently, gefitinib is marketed in several countries in eastern Asia and in the late 2009 was approved by EMEA for the treatment of locally advanced or metastatic NSCLC patients who have been pretreated with platinum-based chemotherapy.

Two further studies retrospectively analyzed gefitinib in previously treated elderly patients with advanced NSCLC and reported a response rate of 0% and 5%, respectively. However, in the Cappuzzo et al. study, the stable disease rate was 45% [26, 27]. 

Recently, the INTEREST-randomized phase III trial in previously treated NSCLC established noninferior survival of gefitinib compared with docetaxel (7.6 versus 8.0 months, resp.), suggesting that gefitinib is a valid treatment for pretreated patients with advanced NSCLC. Superiority of gefitinib in patients with high EGFR-gene-copy number was not proven [28]. In our knowledge, the only published paper on gefitinib as first-line treatment and elderly patients with advanced NSCLC was published by Crinò et al. [29]. They performed a randomized phase II study (INVITE) of gefitinib versus vinorelbine in 196 chemotherapy-naïve unselected elderly patients (age ≥70 years). Patients enrolled in this study reflected a European population seen in clinical practice, because the vast majority of patients were male (77%), were smokers (82%), and had squamous cell carcinoma (48%). The primary end point was progression-free survival (PFS). Secondary end points were overall survival (OS), objective response rate (ORR), quality of life (QoL), pulmonary symptom improvement (PSI), and tolerability. This study showed no statistical difference between gefitinib and vinorelbine in terms of PFS (2.7 versus 2.9 months, resp. *P* = .310), OS (5.9 versus 8.0 months, resp.), ORR (3.1% versus 5.1%, resp.), and disease control rates (43.3% versus 53.5%, resp.); however, the toxicity profile and overall QoL assessments favored gefitinib. Drug-related serious adverse events (AEs) were less frequent in the gefitinib arm versus vinorelbine arm (12.8% versus 41.7%, resp.). Patients treated with gefitinib had a numerically lower incidence of fatigue and gastrointestinal AEs, notably constipation, which is an important side effect in the elderly population and also hematologic toxicity was confined to patients treated with vinorelbine. Most patients were analyzed for EGFR gene copy number by FISH, and surprisingly, those who were EGFR FISH-positive and who received gefitinib appeared to have poorer outcomes than those who were EGFR FISH-negative and who received gefitinib. In the small subgroup of EGFR FISH-positive patients, those treated with vinorelbine achieved nonsignificant better PFS and OS than those treated with gefitinib. A clear explanation for the discrepancy in FISH result is not currently evident, but it could be useful to perform EGFR as well K-RAS mutation analyses in this patient population. Unfortunately, in INVITE trial there were too few patients in the K-RAS mutation analysis to draw any accurate conclusions [29] (Table 1). 

The first-line gefitinib versus carboplatin/paclitaxel (Iressa Pan-Asia Study (IPASS) study was a phase III study in clinically selected patients in East Asia who had advanced NSCLC. The primary end point was PFS and evaluations of efficacy according to the baseline biomarker status of EGFR were planned exploratory objectives. There was a significant interaction between treatment and EGFR mutation with respect to PFS (*P* < .001). PFS was significantly longer among patients receiving gefitinib than that among those receiving carboplatin-paclitaxel in the mutation-positive subgroup (*P* < .001) and significantly shorter among patients receiving gefitinib than that among receiving carboplatin-paclitaxel in the mutation-negative subgroup (*P* < .001). The ORR in the overall population was significantly higher with gefitinib than that with carboplatin-paclitaxel (43% versus 32.2%, resp.; *P* < .001). The ORR was 71.2% with gefitinib versus 47.3% with carboplatin-paclitaxel in the mutation-positive subgroup (*P* < .001) and 1.1% versus 23.5%, respectively, in the mutation-negative subgroup (*P* = .001). Significantly more patients in the gefitinib group than in the carboplatin-paclitaxel group had a clinically relevant improvement in quality of life (*P* < .001). Interstizial lung disease (ILD) events occurred in 2.6% patients treated with gefitinib and in 1.4% patients treated with carboplatin-paclitaxel. The Authors concluded that gefitinib is superior to carboplatin-paclitaxel as an initial treatment for pulmonary adenocarcinoma among nonsmokers or former light smokers in East Asia. The presence in the tumor of a mutation of the EGFR gene is a strong predictor of a better outcome with gefitinib. This study was not targeted for elderly NSCLC patients (median age of enrolled patients was 57 years); however it showed that patients in whom an EGFR mutation has been identified will benefit most from first-line therapy with gefitinib [30].

## 3. Erlotinib

Erlotinib in a phase III, randomized, placebo-controlled trial (BR.21 study) has been proven to prolong survival compared with best supportive care (6.7 months versus 4.7 months for erlotinib and for placebo, resp.) in unselected NSCLC patients after first- or second-line chemotherapy failure [31]. Based on the results of this trial, erlotinib was approved by the US FDA in November 2004 and by the EMEA in October 2005 for the treatment of chemotherapy-resistant advanced NSCLC patients. However, the mild safety profile of erlotinib makes it particularly suitable to be tested prospectively as a single agent in the first-line treatment of elderly patients as an alternative to cytotoxic chemotherapy.

Wheatley-Price et al. performed a retrospective analysis of elderly subgroup enrolled in BR.21 study and demonstrated that elderly patients treated with erlotinib gain similar survival and QoL benefits as younger counterpart but experience greater toxicity. At study entry, 162 patients (22%) were pretreated elderly (112 randomly assigned to erlotinib). Eight percent of elderly erlotinib patients had an objective response, and 70% had a best response of stable disease compared with 9% and 59% of young patients, but these differences were not significant. No significant differences were seen in median duration of response (39 weeks versus 34 weeks, resp.) and in survival benefit between elderly and young patients (3.0 months versus 2.1 months, resp.). Thirty-five % of the elderly group experienced severe toxicity (grade  ≥3) compared with 18% of the younger group (*P* < .001). The elderly were more likely than the younger group to have grade  ≥3 rash, fatigue, stomatitis, and dehydration, as well as any grade of anorexia and fatigue. Fatal drug-related toxicities were unusual and occurred in only five patients (two young and three elderly patients). When we consider dyspnea, cough, and pain, QoL benefits were similar in elderly and young patients [32] (Table 1).

A recent phase II study by Jackman et al. reported the efficacy of erlotinib as first-line treatment in 80 unselected patients greater than 70 years of age with stage IIIB or IV NSCLC. There were eight partial responses and thirty-three stable diseases for an overall disease control rate of 51%. The most frequent AEs were rash (79%) and diarrhea (69%). In general, toxicities were mild and easily managed. Fifteen patients experienced treatment-related toxicities ≥grade 3. Twelve patients were removed from the protocol because of erlotinib-related toxicity (three patients with ILD, three with dehydration, three with diarrhea, one with hemoptysis, one with rash, and one with anorexia). There were four patients with possible ILD, with one treatment-related death. Median time to progression (TTP) was 3.5 months and median survival time was 10.9 months. The 1- and 2-year survival rates were 46% and 19%, respectively. Treatment-related rash was correlated with prolonged TTP and survival, while smoking history and weight loss at presentation were predictors of shorter survival. The presence of an EGFR mutation was strongly correlated with disease control, prolonged TTP (*P* < .017) and survival (*P* < .027). Not surprisingly, patients with a KRAS mutation had no clinical responses and a median TTP of only 2.5 months. Finally, tumor histology (64% of patients had adenocarcinoma) was not associated with improved survival in this trial [33] (Table 1). Hesketh et al. published the results of phase II study that evaluated the efficacy and tolerability of single-agent erlotinib in 81 unselected chemotherapy-naïve patients with advanced NSCLC and a PS of 2. The median age of enrolled patients was 74 years. The overall control disease rate was 42% and the median survival of 5 months is comparable with that reported in prior trials employing chemotherapy alone in the PS 2 population [34–38]. This SWOG trial demonstrated that single-agent erlotinib resulted in an acceptable but significant level of treatment-related side effects for a substantial minority of chemotherapy-naive patients with advanced NSCLC and PS 2. Moreover, erlotinib does not offer a significant treatment advance over chemotherapy in unselected PS 2 patients [38]. Lilenbaum et al. confirmed that unselected patients with advanced NSCLC and PS 2 are best treated with combination chemotherapy as first-line therapy. However, erlotinib may be considered in patients selected by clinical or molecular markers [37].

A new paradigm of utilizing TKIs is as maintenance drug after first-line treatment obtaining control disease as recently showen in SATURN and ATLAS studies. The SATURN trial investigated the role of maintenance therapy with erlotinib in EGFR IHC-positive patients. Erlotinib significantly increased PFS (*P* = .000003) and overall survival from 11 months to 12 months compared with placebo [39]. In ATLAS trial, an improvement in PFS was obtained with the combination of erlotinib and bevacizumab versus bevacizumab and placebo as a maintenance therapy [40]. Moreover, in TORCH trial, Gridelli et al. are investigating whether erlotinib as first-line therapy until progression followed by chemotherapy with cisplatin/gemcitabine will not be inferior in terms of survival to the standard arm, consisting of first-line cisplatin/gemcitabine for 6 cycles, followed at progression by erlotinib until second progression [41].

## 4. Clinical and Molecular Predictors for Response to EGFR TKIs

Clinical data suggest that EGFR TKIs gefitinib and erlotinib are more active in certain NSCLC histotypes, such as in adenocarcinomas and bronchioloalveolar carcinomas (BAC), in women, in never smokers and in Asian ethnicity patients [30, 31, 42–46]. However, in BR.21 trial, survival was significantly improved in all subgroups of patients receiving erlotinib versus placebo, such as male smokers with squamous cell carcinoma [31]. A subgroup analysis of the TRIBUTE trial showed that the addition of erlotinib to paclitaxel/carboplatin prolonged survival in patients who never smoked (median survival 22.5 versus 10.1 months, *P* = .01) [47]. Skin rash is a common adverse effect observed in all clinical trials with EGFR-targeting agents. The incidence of rash was higher with erlotinib than gefitinib [48] and may be due to the lower plasma concentration of gefitinib compared with erlotinib when administered at the recommended dosages of 250 and 150 mg/day, respectively. A correlation between the severity of skin rash (grade ≥2) and significant improvement of survival was observed in several clinical trials [49–51], and therefore, skin rash seemed to function as a surrogate marker of efficacy [51].

Somatic mutations in the EGFR gene are most frequently detected in NSCLC patients with a better outcome, including adenocarcinomas histology, in particular BAC, nonsmokers, females, and Asian ethnicity [37, 52, 53]. The most common mutations of EGFR are in a frame deletion in exon 19 (45%–50% of all somatic EGFR mutations) and a missense mutation leading to leucine to arginine substitution at codon 858 (L858R) in exon 21 (35%–45% of mutations) [54]. Emerging data suggest that patients with NSCLC and EGFR exon 19 deletion have a longer survival following treatment with gefitinib or erlotinib compared with those with L858R mutation [55–57]. Recently, IPASS study showed that, in the subgroup of 261 patients who were positive for the EGFR mutation, PFS was significantly longer among those receiving gefitinib that than among those receiving carboplatin-paclitaxel as first-line treatment (*P* < .001) [37]. Several retrospective analyses of clinical trials have failed to demonstrate the correlation between EGFR IHC status and response, TTP and OS in NSCLC patients treated with gefitinib or erlotinib [58–62]. Conversely, in other clinical trials, it has been shown that high levels of EGFR protein expression are associated with response and improvement of survival [63–66]. Of note, EGFR FISH-positive status was significantly associated with certain clinical and biological characteristics predictive for TKI sensitivity, such as female sex, never-smoking history, and the presence of EGFR mutations [25]. However, the most predictive marker of response remains EGFR mutation/deletion status in the kinase domain.

In approximately 15%–30% of lung adenocarcinomas, activating mutations in the RAS family member were found. This more commonly occurs in patients with smoking history and these mutations are most frequently recorded in codons 12 and 13 in exon 2 of the K-RAS gene [67–69]. The role of K-RAS mutation in NSCLC patients is still controversy, but it seems associated with a worse outcome and a shorter survival [70]. 

## 5. Discussion

Treatment of elderly patients who have NSCLC remains a challenge. Older adults represent a heterogenous population, despite similar chronologic age. Individualizing treatment decision-making based on careful patient assessment is currently an active area of research in geriatric oncology and will hopefully lead to improved treatment outcomes for older adults. These patients have more comorbidities and tend to be more intolerant of toxic medical treatment than their younger counterparts [71]. A comprehensive geriatric assessment (CGA), which has proven to provide more indications compared with the performance status assessment alone, ought to be carried out. The CGA should include evaluation of comorbidities, socioeconomic issues, nutritional status, polypharmacy, functional dependence, emotional and cognitive conditions, an estimate of life expectancy, and recognition of frailty. Nevertheless, a CGA may be too lengthy in busy clinical practice; so, validated and shorter screening instruments are needed. The Cardiovascular Health Study divided elderly patients into three groups (fit, prefrail, frail) according to five items (unintentional weight loss, self-reported exhaustion, weakness, walking speed, and level of physical activity) and has gained particular prominence because it is well correlated with mortality and risk of functional dependence [72, 73]. However, actually CGA remains the best option to decide on the best-suited treatment modality for a particular geriatric patient. Based on prospective trials for unselected elderly advanced NSCLC, single-agent chemotherapy with third-generation agents (vinorelbine, gemcitabine, taxanes) is still considered the recommended treatment.

Among targeted therapies, the EGFR TKIs, erlotinib and gefitinib are the most promising agents and have been shown in phase II trials to be active and well tolerated as first-line treatment of advanced NSCLC in the elderly. In responders to EGFR TKIs, the symptom relief was dramatically obtained also in PS ≥  2 patients [74–76]. Of note, most patients at the time of recurrence of NSCLC have suffered some types of toxicity from previous chemotherapy or comorbidities such as chronic renal failure, contraindicating any further chemotherapy. In a Gridelli et al. report, erlotinib at full dosage was administered to three advanced NSCLC patients unsuitable for chemotherapy because of chronic renal failure. Further renal function was not impaired by therapy with erlotinib, and no severe toxicity was recorded. Erlotinib is metabolized in the liver, mainly by the cytochrome p-450 isoenzyme CYP 3A4. Erlotinib and its metabolites are excreted predominantly via feces, with renal elimination of drug and metabolites accounting for less than 9% of the administered dose [77]. With interest are awaited the results of the GEST phase II study, where elderly patients with untreated advanced NSCLC were randomized to receive sorafenib plus gemcitabine or sorafenib plus erlotinib [78]. Another study currently ongoing is the ZELIG phase II randomized study with vandetanib plus gemcitabine versus gemcitabine alone in the same subset of elderly patients. 

Finally, elderly patients with advanced NSCLC and carriers of an EGFR mutation may be considered for gefitinib or erlotinib as first-line treatment. However, further specifically designed phase III randomized trials are needed to optimize medical treatment of elderly patients with advanced NSCLC.

## Figures and Tables

**Table 1:** Main studies on EGFR TKIs for elderly (≥70 years) patients with advanced NSCLC.

Author	Study arm	No. of patients	Response rate (%)	Median OS (months)
Shepherd* et al. [31]	Erlotinib versus	112	8	7.6
	Placebo	50	0	5.0
Wheatley-Price^§^ et al. [32]	Erlotinib	80	10	10.9
Crinò^§^ et al. [29]	Gefitinib versus	99	3.1	5.9
	Vinorelbine	97	5.1	8.0

*2nd or 3rd -line; ^§^first -line.
